# Toward Aerogel Electrodes of Superior Rate Performance in Supercapacitors through Engineered Hollow Nanoparticles of NiCo_2_O_4_

**DOI:** 10.1002/advs.201700345

**Published:** 2017-11-08

**Authors:** Jianjiang Li, Shuai Chen, Xiaoyi Zhu, Xilin She, Tongchao Liu, Huawei Zhang, Sridhar Komarneni, Dongjiang Yang, Xiangdong Yao

**Affiliations:** ^1^ Collaborative Innovation Center for Marine Biomass Fibers Materials and Textiles of Shandong Province School of Environmental Science and Engineering Qingdao University Qingdao 266071 P. R. China; ^2^ Shanghai Key Lab of Electrical Insulation and Thermal Aging Shanghai Jiao Tong University Shanghai 200240 P. R. China; ^3^ State Key Laboratory of Coal Conversion Institute of Coal Chemistry Chinese Academy of Science Taiyuan 030001 P. R. China; ^4^ School of Advance Materials Peking University Shenzhen Graduate School Peking University Shenzhen 518055 P. R. China; ^5^ College of Chemical and Environmental Engineering Shandong University of Science and Technology Qingdao 266590 P. R. China; ^6^ Materials Research Institute and Department of Ecosystem Science and Management the Pennsylvania State University University Park PA 16802 USA; ^7^ Queensland Micro‐ and Nanotechnology Centre (QMNC) Griffith University Nathan, Brisbane Queensland 4111 Australia

**Keywords:** aerogels, hollow structure, nickel cobaltite, seaweed, supercapacitor

## Abstract

A biomass‐templated pathway is developed for scalable synthesis of NiCo_2_O_4_@carbon aerogel electrodes for supercapacitors, where NiCo_2_O_4_ hollow nanoparticles with an average outer diameter of 30–40 nm are conjoined by graphitic carbon forming a 3D aerogel structure. This kind of NiCo_2_O_4_ aerogel structure shows large specific surface area (167.8 m^2^ g^−1^), high specific capacitance (903.2 F g^−1^ at a current density of 1 A g^−1^), outstanding rate performance (96.2% capacity retention from 1 to 10 A g^−1^), and excellent cycling stability (nearly without capacitance loss after 3000 cycles at 10 A g^−1^). The unique structure of the 3D hollow aerogel synergistically contributes to the high performance. For instance, the 3D interconnected porous structure of the aerogel is beneficial for electrolyte ion diffusion and for shortening the electron transport pathways, and thus can improve the rate performance. The conductive carbon joint greatly enhances the specific capacity, and the hollow structure prohibits the volume changes during the charge–discharge process to significantly improve the cycling stability. This work represents a giant step toward the preparation of high‐performance commercial supercapacitors.

As new and promising energy‐storage devices, supercapacitors (SCs) have attracted tremendous research attention in recent years because of their high power densities, short charging/discharging time, excellent cycling stability, and electrochemical reversibility.^1^, ^2^, ^3^ Particularly, transitional metal oxide (TMO) electrodes possess multiple oxidation states that enable rich redox reactions for excellent pseudocapacitance and thus have attracted wide research attention in recent years.^4^, ^5^, ^6^, ^7^, ^8^ As a typical TMO electrode material, spinel nickel cobaltite (NiCo_2_O_4_) is attracting much attention due to its environmentally benign nature and high theoretical capacity (higher than 2000 F g^−1^).^9^, ^10^ Compared with the two corresponding single component oxides (NiO, Co_3_O_4_), NiCo_2_O_4_ possesses a much higher electronic conductivity and electrochemical activity. However, for the nature of a conductor, the conductivity of NiCo_2_O_4_ still needs to be further improved to achieve higher performance. Therefore, most of the reported NiCo_2_O_4_ electrodes for high‐performance SCs so far are based on various NiCo_2_O_4_ nanostructures grown on conductive substrates by an in situ growth method.^11^ The conductivity of the electrode can be enhanced greatly by the conductive substrates such as metal Ni foam and graphitic carbon.^3^, ^6^, ^12^ However, the in situ synthesis approach is not convenient for a large‐scale production.

The NiCo_2_O_4_ electrode also suffers from poor ion transport kinetics and therefore, would lead to bad rate performance.^13^, ^14^ Recently, NiCo_2_O_4_ hollow nanoparticles (HNPs) have been developed as electrodes for SCs to enhance the performance since they can provide high specific surface area and short ion diffusion path. However, these nanostructures always result in increased number of grain boundaries, which could increase electrical resistance in a solid electrode. Also, the synthesis of NiCo_2_O_4_ HNPs was generally dependent on hard‐template method which is limited for practical applications due to its high cost, inconvenient, and multistep synthesis.^15^, ^16^, ^17^ To address the above problems, it is feasible to develop a cost‐effective pathway for the synthesis of NiCo_2_O_4_ HNPs and assemble them into a matrix with interconnected porosity and excellent electrical conductivity. Aerogels, a special class of porous solid materials with hierarchical 3D morphology and adjustable porosity, precisely meet the above requirements. It could be expected that a 3D NiCo_2_O_4_ aerogel, which is composed of HNPs of NiCo_2_O_4_ connected with conductive joint, can combine the merits of each assembling unit to synergistically enhance the intrinsic properties of each component such as electrical conductivity and ion transport kinetics.

In this work, NiCo_2_O_4_@carbon aerogels assembled by HNPs of NiCo_2_O_4_ and nanocarbon joint were prepared by using sustainable sodium alginate (SA) as template and carbon source. The method reported here involves the synthesis of NiCo‐alginate hydrogel and aerogel, and subsequent pyrolysis in N_2_ and oxidation in air. The special “egg‐box” structure formed in SA after ion‐exchange with Ni^2+^ and Co^2+^ cations is the key to guide the synthesis.^12^, ^18^, ^19^, ^20^ The “egg‐box” structure is also essential to control the diameter of NiCo_2_O_4_ HNPs within the nanoscale. This facile and cost‐effective method is more suitable for large scale production in comparison with the complicated hard‐template synthesis. The composite aerogels were designed and synthesized by integrating the 3D porous structure and functional components. When evaluated as electrode materials for SCs, these NiCo_2_O_4_@carbon aerogels displayed high capacitance (903.2 F g^−1^ at a current density of 1 A g^−1^), outstanding rate performance (96.2% capacity retention from 1 to 10 A g^−1^), and superior cycling stability (nearly without capacitance loss after 3000 cycles at 10 A g^−1^).

Our strategy for the synthesis of the 3D NiCo_2_O_4_@carbon aerogels is illustrated in **Figure** **1**. It is a typical hydrogel/aerogel conversion process in the presence of SA as template and carbon source.^21^ As shown in Figure 1a, SA is a kind of poly natural polysaccharide which is composed of β‐d‐mannuronate (M) and α‐l‐guluronate (G) monomers. The G blocks of SA are active to coordinate with divalent or trivalent cations to form “egg‐box” structure.^20^, ^22^ In the first step, the aqueous solution (brick red color) of mixed Ni^2+^ and Co^2+^ cations with Ni/Co molar ratio of 1:2 was dropped into an aqueous solution of SA to prepare NiCo‐alginate hydrogel (Figure 1a). During the ion‐exchange progress, Na^+^ cations in SA chain were exchanged by Ni^2+^ and Co^2+^ cations. The gelation was due to the formation of “egg‐box” structure where the Ni^2+^ and Co^2+^ cations were confined by four G blocks of SA (Figure 1b).^20^, ^22^ This step was also the key to control the ratio of active NiCo_2_O_4_ NPs and carbon joint in the final 3D NiCo_2_O_4_ aerogels by adjusting the concentration of Ni^2+^ and Co^2+^ cations. In the second step, the obtained NiCo‐alginate hydrogels (Figure 1b) were converted to NiCo‐aerogels via freeze drying process. The color of NiCo‐aerogels changed from brick red to blue after the freeze‐drying process (Figure 1c). This change was attributed to the loss of water of hydration from Ni^2+^/Co^2+^ ions. A 3D porous framework in the aerogels was formed at this stage by the removal of the adsorbed water around the alginate macromolecules. Finally, the “egg‐box” confined Ni^2+^/Co^2+^ cations could be first converted to NiCo alloy@carbon core/shell NPs by pyrolysis in N_2_, and then the core/shell NPs were converted to NiCo_2_O_4_ HNPs through the typical nanoscale Kirkendall effect (Figure 1c).^21^, ^23^ In addition, the amorphous carbon was consumed, and only graphitic nanocarbon was left behind to connect the NiCo_2_O_4_ HNPs.

**Figure 1:**
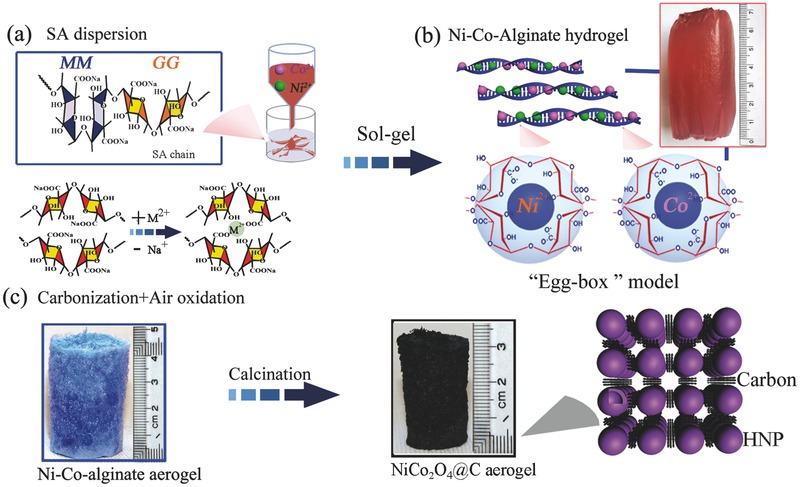
Schematic illustration for the fabrication of NiCo_2_O_4_ aerogel.

Powder X‐ray diffraction (PXRD) was carried out to monitor the evolution process of the NiCo‐alginate aerogels after pyrolysis and oxidation treatment. Figure S1 (Supporting Information) shows the XRD pattern of NiCo‐alginate aerogels after the pyrolysis process. Three diffraction peaks at 44.35°, 51.69°, and 76.12° are observed from the XRD patterns, which are ascribed to Ni–Co alloy (JCPDS No. 97‐010‐8308). The intensity of the peaks increased with increasing Ni, Co content. After oxidation in air at 300 °C, the diffraction peaks of the cubic spinel NiCo_2_O_4_ (JCPDS No. 73‐1702) were observed from the patterns (Figure S2, Supporting Information), i.e., the NPs of NiCo alloy were oxidized to NiCo_2_O_4_ NPs. According to the Raman spectra shown in Figure S3 in the Supporting Information, besides the vibration peaks assigned to F_2g_, E_g_, F_2g_, and A_1g_, modes of NiCo_2_O_4_,^24^ two prominent characteristic peaks of carbon, D band at 1364.9 cm^−1^ and G band at 1592.5 cm^−1^ were observed.^10^, ^21^ The carbon species resulted from the Ni‐Co‐catalyzed carbonization of C–H–O framework of alginate “egg‐box”. The two peaks at 1364.9 and 1592.5 cm^−1^ are assigned to the characteristic D (defects and disorder) and G (graphitic) bands of carbon materials, respectively.^25^ The intensity ratio of D/G (*I*_D_/*I*_G_) value demonstrates the extent of structural disorder of carbon materials and the *I*_D_/*I*_G_ values of the NiCo_2_O_4_ NPs here are in the range of 0.85–0.92, indicating that the formed carbon material possessed high graphitic degree and excellent electroconductivity. Field emission scanning electron microscopy (FESEM) was conducted to determine the morphology and microstructure of the obtained NiCo_2_O_4_ aerogel. As shown in **Figure** **2**a, the Ni‐Co‐12 aerogel, which was prepared with an aqueous solution (total cation concentration of 12 mmol L^−1^), was composed of uniform NiCo_2_O_4_ NPs with a diameter of 30–40 nm. More importantly, the sample exhibited highly interconnected 3D porous structure. A cross‐section image of the Ni‐Co‐12 aerogel revealed that the 3D porous network was throughout the whole sample (the inset of Figure 2a). The unique 3D porous network is amenable to enhance the specific capacitance and high rate performance of SCs, since the different types of pores are beneficial for the storage of much electrolyte and for the rapid diffusion and transport of reactive molecules, ions, and electrolyte. The FESEM images of Ni‐Co‐3, Ni‐Co‐6, and Ni‐Co‐18 aerogels are shown in Figure S4 in the Supporting Information. Similar to Ni‐Co‐12, the 3D porous network morphology can be observed from all the samples. Compared to other samples, larger NiCo_2_O_4_ HNPs could be seen in the Ni‐Co‐18 aerogel (Figure S4d, Supporting Information) which is due to the treatment of higher concentration of Ni^2+^ and Co^2+^ ions. With the increased concentration of Ni^2+^ and Co^2+^ used during ion‐exchange, the larger NiCo_2_O_4_ particles were generated during the air oxidation process owing to the Ni^2+^ and Co^2+^ not fully utilized by the SA chains. The above formation of larger NiCo_2_O_4_ particles led to lower Brunauer‐Emmett‐Teller (BET) specific surface area as determined by the adsorption–desorption of N_2_.

**Figure 2:**
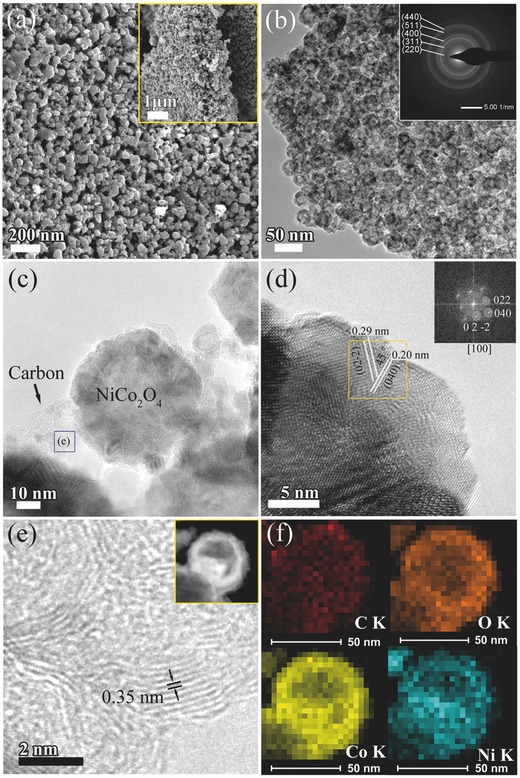
a) SEM image. b–e) HRTEM images. f) The HAADF‐STEM image (the inset in Figure 2e) and EDS elemental mapping of the NiCo_2_O_4_ aerogel (sample: Ni‐Co‐12).

The microstructure of Ni‐Co‐12 aerogel was also investigated by transmission electron microscopy (TEM) and high resolution TEM (HRTEM). The contrast (dark/bright) between the boundary and center of the NiCo_2_O_4_ NPs suggested their typical hollow nature (Figure 2b). The bright rings in the selected‐area electron diffraction pattern (the inset in Figure 2b) could be well indexed to the cubic spinel structure and also showed the polycrystalline characteristics of NiCo_2_O_4_ aerogel in accordance with the XRD results. The TEM image of a single NiCo_2_O_4_ HNP is displayed in Figure 2c. The diameter of the NiCo_2_O_4_ HNP is ≈40 nm and the shell thickness is ≈10 nm. The HRTEM image of the selected area of the NiCo_2_O_4_ HNP (Figure 2d) indicates that the NiCo_2_O_4_ HNP is well crystallized with perfectly aligned lattice fringes across the crystals. Two sets of lattice fringe spacings of 0.20 and 0.29 nm are assigned to the (040) and (02‐2) planes of face centered cubic NiCo_2_O_4_, respectively. In addition, we can clearly see that the NiCo_2_O_4_ HNPs were connected with graphitic carbon joint with a lattice fringe space of 0.35 nm (Figure 2e), which is in good agreement with the results of Raman spectra (Figure S3, Supporting Information). These graphitic carbon joints can efficiently enhance the electrical contact between the NiCo_2_O_4_ HNPs and thus improve their stability upon charge/discharge cycling. Furthermore, the energy dispersive X‐ray spectroscopy (EDS) spectrum of Ni‐Co‐12 aerogel (Figure S5, Supporting Information) shows the signals of Ni, Co, and O elements and the molar ratio between Co and Ni is nearly equal to 2. The high‐angle annular dark‐field scanning transmission electron microscopy (HADDF‐STEM) image (the inset in Figure 2e) and EDS elemental mappings (Figure 2f) show that the C, O, Ni, and Co atoms have a homogeneous distribution in a single NiCo_2_O_4_ HNP.

The N_2_ adsorption/desorption isotherms and the pore size distribution of the NiCo_2_O_4_ aerogels are shown in Figure S6 in the Supporting Information. The major N_2_ adsorption of all the samples occurs at a low relative pressure (*P*/*P*_0_ < 0.02) but rises slowly thereafter to an almost plateau and at higher relative pressures shows an obvious hysteresis loop, which is typical of type IV adsorption/desorption isotherms indicating the existence of mesoporosity. The specific surface area and pore volume of all the samples are presented in Table S1 in the Supporting Information. The specific surface areas of the Ni‐Co‐3, Ni‐Co‐6, Ni‐Co‐12, and Ni‐Co‐18 aerogels are determined to be 77.5, 121.4, 167.8, and 137.6 m^2^ g^−1^, respectively. The insets of Figure S6 (Supporting Information) show the pore size distribution curves of all the samples, which indicate mesopores in the 3–4 nm and 15–40 nm ranges. The pore size distribution of Ni‐Co‐12 (the inset of Figure S6c, Supporting Information), in particular, shows the narrowest pore size distribution of 3–20 nm and consequently the highest specific surface area. Therefore, the Ni‐Co‐12 aerogel is selected for further investigation. The porosity derived from the aerogel structure apparently can be used as good current collector in energy storage applications and hence they are expected to be useful for electrochemical applications.^26^

The detailed chemical composition and bonding configurations of Ni‐Co‐12 aerogel with the highest surface area were further studied by X‐ray photoelectron spectroscopy (XPS) analysis (**Figure** **3**). The survey spectrum (Figure 3a) indicates the presence of Ni, Co, O, and C elements only, as expected. By using a Gaussian fitting method, the Ni_2p_ emission spectrum (Figure 3b) is best fitted with two spin‐orbit doublets $\left({\mathrm{Ni}}_{2p}^{1/2}\;\;,\;{\mathrm{Ni}}_{2p}^{3/2}\;\;\right)$, which are characteristic of Ni^2+^ and Ni^3+^ with two satellites peaks. The peaks at 855.7 and 873.1 eV are indexed to Ni^2+^, while the other peaks at 854.2 and 871.7 eV are ascribed to Ni^3+^. Similarly, in Co_2p_ emission spectrum (Figure 3c), two kinds of Co species are observed, which can be assigned to Co^2+^ and Co^3+^. Specifically, the peaks at 780.7 and 795.8 eV are ascribed to Co^2+^ while the other two peaks at 779.4 and 794.6 eV belong to Co^3+^. Thus, the XPS results demonstrated that the electron couples of Ni^3+^/Ni^2+^ and Co^3+^/Co^2+^ coexisted in NiCo_2_O_4_ aerogel, which is consistent with the previously reported results for NiCo_2_O_4_.^10^ The C 1s emission spectrum (Figure 3d) can be divided into four peaks centered at 284.6, 286.4, 287.8, and 289.0 eV, which is attributed to the C—C/C=C, C—O, C=O, and O=C—O groups,^6^ respectively. This also indicates that the presence of graphitic carbon joints in NiCo_2_O_4_ aerogel.

**Figure 3:**
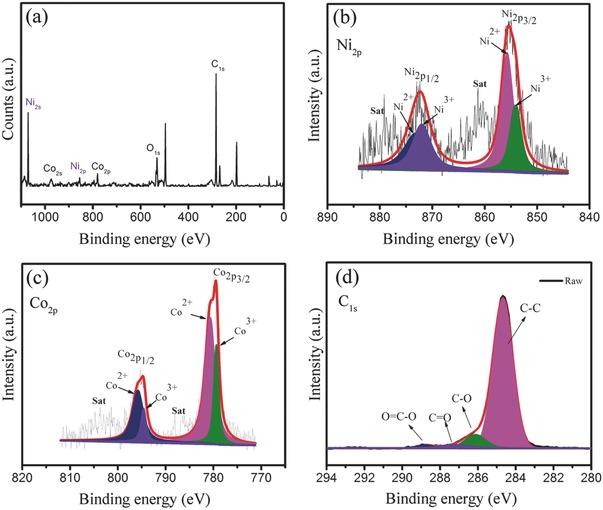
XPS spectra of Ni‐Co‐12 aerogel: a) survey spectrum, b) Ni_2p_, c) Co_2p_, and d) C_1s_.

To study the electrochemical performance of all NiCo_2_O_4_ aerogels, the cyclic voltammetry (CV) and galvanostatic charge–discharge (GCD) measurements were performed as shown in **Figure** **4** and Figure S7 (Supporting Information). Figure 4a shows typical CV curves of Ni‐Co‐12 aerogel measured at various sweep rates ranging from 5 to 50 mV s^−1^ in a potential range of 0.0–0.6 V (vs Hg/HgO) in 6.0 m KOH solution. A pair of well‐defined redox peaks are clearly observed from all the CV curves, which mainly originated from the reversible faradic redox reaction related to M—O/M—O—OH (M = Ni or Co).^27^ Apparently, when the sweep rate is increased, the current density increases, and the position of anodic peak shifts positively to higher potential due to polarization but keeping similar curve shape,^28^ indicating that the fast and reversible redox reactions occur at the electrode/electrolyte interface.^29^, ^30^ Even at 50 mV s^−1^ scan rate (Figure 4a), the CV curve still shows a pair of redox peaks, since the carbon joints connected to the hollow NiCo_2_O_4_ NPs are beneficial to hopping electrons and could accelerate redox reactions.^4^ To investigate the specific capacitance and the rate performance of the Ni‐Co‐12 aerogel, GCD measurements were performed in a 6.0 m KOH solution at different current densities. Figure 4b presents the galvanostatic charge–discharge voltage curves of the NiCo_2_O_4_ electrode at different current densities ranging from 1 to 10 A g^−1^. It can be observed from Figure 4b that there are voltage plateaus at around 0.35 V, which is consistent with the above CV results. The specific capacitances of the Ni‐Co‐12 were calculated to be 903.2, 898.7, 883.0, 874.5, 870.0, 869.0 F g^−1^ at the current densities of 1, 2, 4, 6, 8, and 10 A g^−1^, respectively. Also, the CV and GCD curves of Ni‐Co‐3, Ni‐Co‐6, and Ni‐Co‐18 are presented in Figure S7 in the Supporting Information. The calculated specific capacitance of all samples is summarized in Table S2 in the Supporting Information. The specific capacitance values of Ni‐Co‐3, Ni‐Co‐6, Ni‐Co‐12, and Ni‐Co‐18 aerogels are 617.8, 670.4, 903.2, and 763.0 F g^−1^ at the current density of 1 A g^−1^. Their capacitance retentions are 94.6, 94.7, 96.2, and 91.1% when the current density rises to 10 A g^−1^, respectively. It is well‐known that the specific capacitance of electrode materials is highly affected by their specific surface area and pore structures. The specific capacitance values determined here (Table S2, Supporting Information) are also consistent with the specific surface areas of different aerogels (Table S1, Supporting Information). As a comparison, the SEM image and electrochemical performance of nonporous NiCo_2_O_4_ is shown in Figure S8 in the Supporting Information. It can be seen that nonporous NiCo_2_O_4_ has larger size than that of the NiCo_2_O_4_ aerogel. Its specific capacitance is ≈580.0 F g^−1^ at the current density of 1 A g^−1^, much lower than that of Ni‐Co‐12. Figure S9 (Supporting Information) shows a comparison of the specific capacitance of Ni‐Co‐12 aerogel with those of other materials previously reported.^6^, ^24^, ^27^, ^31^, ^32^, ^33^, ^34^, ^35^, ^36^, ^37^, ^38^, ^39^, ^40^, ^41^ Obviously, our results are much better than those reported by others.

**Figure 4:**
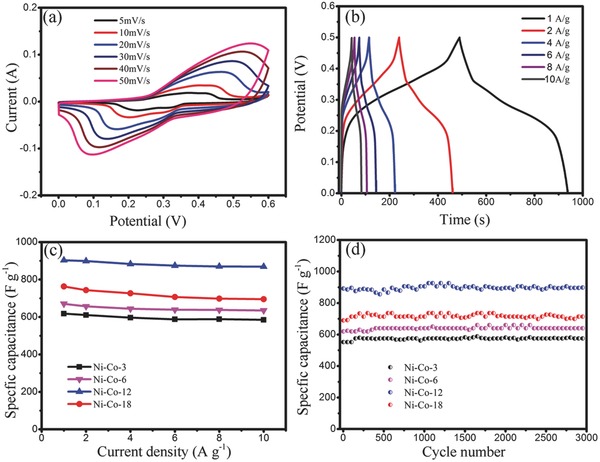
a) CV and b) GCD curves of the Ni‐Co‐12 aerogel sample at different scan rates and different current densities in 6.0 m KOH aqueous solution, respectively. c) Specific capacitances of electrodes of NiCo_2_O_4_ aerogels at different discharge current densities (A g^−1^). d) The cycling performance of NiCo_2_O_4_ aerogels within 3000 cycles at a current density of 10 A g^−1^.

The rate performance of all NiCo_2_O_4_ aerogel is shown in Figure 4c. We can see that all NiCo_2_O_4_ aerogels have excellent rate performance although the corresponding specific capacitance tends to decrease slightly with the increase of current density. Interestingly, the specific capacitances of the Ni‐Co‐12 were about 903.2, 898.7, 883.0, 874.5, 870.0, 869.0 F g^−1^ at the current densities of 1, 2, 4, 6, 8 and 10 A g^−1^, respectively and these results suggest that this NiCo_2_O_4_ aerogel led to an outstanding rate performance with only an ≈6.8% SC loss (from 903 to 869 F g^−1^) by increasing the current density from 1 to 10 A g^−1^. This outstanding rate performance can be attributed to the unique structure of HNP in the Ni‐Co‐12 aerogel, which provided higher specific surface area and thus, more contact interface between the electrolyte ions and the electrode materials. Such aerogel structure can also facilitate the electrolyte ion diffusion and shorten the electron transport pathways, which are beneficial to improve the rate performance of SCs. Figure 4d shows the cycling performance of all the NiCo_2_O_4_ aerogels tested at the current density of 10 A g^−1^. Only a little capacity loss occurred with all the aerogels after 3000 cycles. When the cycle times were extended to 10 000 laps, the capacitance retention of Ni‐Co‐12 is also as high as 92.1% (Figure S10, Supporting Information). Such high capacity retention can be attributed to the unique hollow nanostructures of the aerogels, which provide much space against the volume changes during the charge–discharge process.^42^ Figure S11 (Supporting Information) shows the TEM image of Ni‐Co‐12 after long cycle test. It can be seen that most of the NiCo_2_O_4_ HNPs can retain their hollow structure, indicating super stability of the HNP electrode. Figure S12 (Supporting Information) gives the Nyquist plots of all the samples. Apparently, Ni‐Co‐12 shows the smaller equivalent series resistance (*R*_s_) and charge‐transfer resistance (*R*_ct_) than other three samples, which also leads to better electrochemical performance.

To further investigate electrochemical performance of the NiCo_2_O_4_ aerogel, hybrid supercapacitor (HSC) was assembled. **Figure** **5**a shows the schematic illustration of the HSC, where NiCo_2_O_4_ HNPs and activated carbon were used as the positive and negative electrode, respectively. Figure 5b,c shows the CV and GCD measures of HSC, respectively. The CV curves exhibit obvious oxidation and reduction peaks at 0.9 and 0.8 V due to the transition of Ni^2+^/Ni^3+^ and Co^2+^/Co^3+^. Figure 5d shows the rate performance of HSC at the different current density. The specific capacitance of HSC is 142, 137, 135, 129, 126, and 127 F g^−1^ at the current density of 1, 2, 4, 6, 8 and 10 A g^−1^, respectively. The result is much better than the previous reports (≈74–95 F g^−1^).^43^, ^44^ The Ragone plot of HSC is displayed in Figure S13 in the Supporting Information. An energy density of 71 Wh Kg^−1^ can be achieved at a power density of 1852 W Kg^−1^. Furthermore, the soft package electrochemical device was prepared with NiCo_2_O_4_ aerogel as electrodes using 6.0 m KOH as electrolyte. As shown in Figure S14 in the Supporting Information, the electrochemical devices used Ni‐Co‐12 as electrode were connected in series which powered 19 LED lights (≈1.2 V) and could keep these LEDs bright enough continuously (see Movie S1 in the Supporting Information). Figure S15 (Supporting Information) shows the cycle performance of the above device. It can be seen that the capacitance retention is ≈85.1% after 4000 cycles, which is relative better than the previous report.^45^ Thus, these results clearly demonstrated that the NiCo_2_O_4_ aerogels showed superior supercapacitor behavior.

**Figure 5:**
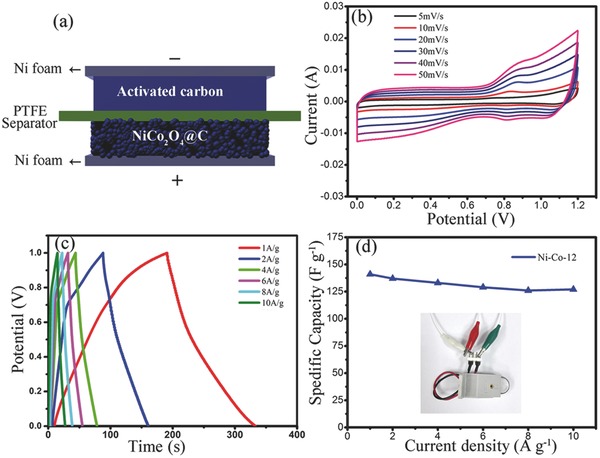
a) Schematic illustration of HSC. b,c) CV and GCD curves of HSC. d) Rate performance of HSC and its digital image (the inset) device.

In conclusion, we have successfully synthesized carbon supported hollow NiCo_2_O_4_ aerogels by a simple metal ion‐exchange method from the seaweed biomass for use in high‐performance supercapacitors. Electrochemical investigations clearly demonstrated that the carbon conjoined hollow NiCo_2_O_4_ aerogels served as excellent electrode materials for electrochemical capacitors with high specific capacitance (903.2 F g^−1^ at 1 A g^−1^), improved rate capability, and excellent cyclic stability even at a high current density of 10 A g^−1^. This biomass‐template pathway is not only facile and cost‐effective for the controlled synthesis of the porous hollow and aerogel structure of NiCo_2_O_4_ but also more suitable for large scale production in comparison with the complicated hard‐template synthesis. So, this synthesis strategy suggests a new pathway to design electrode materials of different chemical compositions for high‐performance supercapacitor.

## Conflict of Interest

The authors declare no conflict of interest.

## Supporting information

Supplementary

Supplementary
