# Supplementary material for: Toward Aerogel Electrodes of Superior Rate Performance in Supercapacitors through Engineered Hollow Nanoparticles of NiCo2O4
Source: Adv Sci (Weinh). 2017 Nov 8;4(12):1700345. doi: 10.1002/advs.201700345 (PMC5737235; doi:10.1002/advs.201700345)
Supplement: Supplementary file 1 — Supplementary [file ADVS-4-na-s001.pdf]

Copyright WILEY-VCH Verlag GmbH & Co. KGaA, 69469 Weinheim, Germany, 2017.

ADVANCED SCIENCE
Open Access

Supporting Information

for *Adv. Sci.,* DOI: 10.1002/advs.201700345

Toward Aerogel Electrodes of Superior Rate Performance in Supercapacitors through Engineered Hollow Nanoparticles of $NiCo_2O_4$

Jianjiang Li, Shuai Chen, Xiaoyi Zhu, Xilin She, Tongchao Liu, Huawei Zhang, Sridhar Komarneni, Dongjiang Yang, and Xiangdong Yao**

Supporting Information for

Towards Aerogel Electrodes of Superior Rate-Performance in Supercapacitors through Engineered Hollow Nanoparticles of $NiCo_2O_4$

Jianjiang Li, Shuai Chen, Xiaoyi Zhu, Xilin She, Tongchao Liu, Huawei Zhang, Sridhar Komarneni, Dongjiang Yang and Xiangdong Yao**

Experimental Section

Synthesis of hollow $NiCo_2O_4$ NPs@Carbon aerogels. All the chemicals were of analytical reagent grade and used as received without further purification. Firstly, 100 mL mixed aqueous $NiCl_2$ and $CoCl_2$ solution with Ni: Co molar ratio of 1: 2 was added drop wise to 200 mL 1.0 wt% aqueous sodium alginate (SA) solution, which resulted in the blue Ni-Co-alginate hydrogels. The as-prepared Ni-Co-alginate hydrogels were freeze-dried, which led to Ni-Co-alginate aerogels. Then, the Ni-Co-alginate aerogels were carbonized at 600 °C under nitrogen flow in a tubular furnace at a heating rate of 5 °C min^{-1} for 2 h followed by additional thermal treatment at 300 °C for 3 h in air atmosphere to get $NiCo_2O_4$ HNPs@Carbon aerogels accroding to the following reaction equation:

$$NiCo_2 + 2O_2 \xrightarrow{300℃} NiCo_2O_4$$

The obtained samples were denoted as Ni-Co-X (X=3, 6, 12 or 18) where X represents the total molar concentration (mmol/L) of Ni^{2+} and Co^{2+} in aqueous solution.

For comparison, the nonporous $NiCo_2O_4$ was also prepared using the seaweed pathway, but the freeze-drying process was not involved.

Characterization. Powder X-ray diffraction (XRD) patterns of the various samples were obtained by a DX2700 diffractometer with Cu Kα radiation (λ=1.5418 Å) at a scan rate of 2° min^{-1}. Raman spectra were recorded using a microscopic confocal Raman spectrometer (Renishaw 1000 NR) upon excitation by 514.5 nm laser light. The specific surface area was calculated by the Brunauer-Emmett-Teller (BET) method from N_2 adsorption data. Pore size distribution plots were derived from the adsorption branch of the isotherms based on the BJH model at 77K. N_2 adsorption/desorption data were collected using an adsorption unit, Tristar 3000 by Micromeritics. The morphology and structure of the samples were investigated by Field emission scanning electron microscope (FESEM; JSM-7001F, JEOL, Tokyo, Japan). Transmission electron microscopy (TEM) and high-resolution transmission electron microscopy (HRTEM) of the as-obtained aerogels were performed on a JEOL JEM-2100F transmission electron microscope at the accelerating voltage of 200kV. X-Ray Photoelectron Spectra (XPS) were collected using a Kratos Axis Ultra DLD electron spectrometer.

Electrochemical measurements. The electrochemical properties of the Ni-Co-X electrodes were first evaluated by using a standard three-electrode cell. The working electrode consisted of active material, carbon black, and polymer binder polyvinylidenedifluoride (PVDF) in a weight ratio of 80:10:10. The slurry was pasted on to Ni foam (1 cm*1 cm)under a pressure of 10 MPa. The loading of active materials on the working electrode is ~ 0.8 mg. The supercapacitor tests were conducted with a CHI 760e electrochemical workstation in an aqueous 6.0 M KOH electrolyte with a three electrode cell where Pt foil served as the counter electrode and Hg/HgO was used as the reference electrode. The cycling stability was conducted by employing the LAND CT2001A battery test system.

The electrochemical property of Ni-Co-12 was further investigated by assembling a hybrid supercapacitor (HSC), where $NiCo_2O_4$ and activated carbon were used as the positive and

negative electrodes, respectively. APTFE film was used as the separator and 6 M NaOH aqueous solution was the electrolyte. The cyclic voltammetry and galvanostatic current charge-discharge were measured on CHI760E electrochemical workstation. EIS measurements were carried out by applying an voltage with 5 mV amplitude in a frequency range from 0.01 Hz to 100 KHz at open circuit potential.

The specific capacitance of the electrodes can be calculated by using the following formula: $C=(I\times\Delta t)/(\Delta V\times m)$ (1)

Where C (F g^{-1}) is the specific capacitance of the electrode, I (A) denotes the discharge current, Δt (s) refers to the discharge time, ΔV (V) is the potential drop during discharge, and m (g) represents the mass of the active material within the electrode.

The energy density (E) and power density (P) were calculated by using the following formula:

$$E=\frac{C\times(\Delta V)^2}{2} \quad (2)$$

$$P=\frac{E}{\Delta t} \quad (3)$$

Where E (Wh kg^{-1}) is the energy density, P (W kg^{-1}) is the power density, ΔV (V) is the cell voltage for charging and discharging, and Δt (h) is the discharge time, respectively.

The symmetry supercapacitor device was designed by using nickel foam as current collector, cellulose membrane and KOH solution as separator and electrolyte, respectively while $NiCo_2O_4$@Carbon aerogel served as the electrode.

Figures

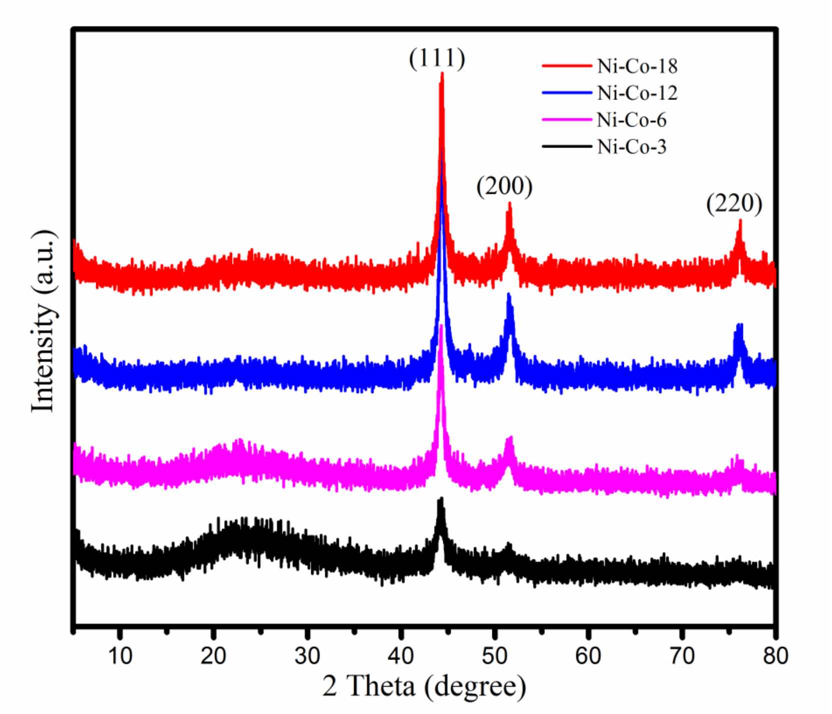

Figure S1. XRD patterns of NiCo-alginate aerogels after carbonization at 600 °C.

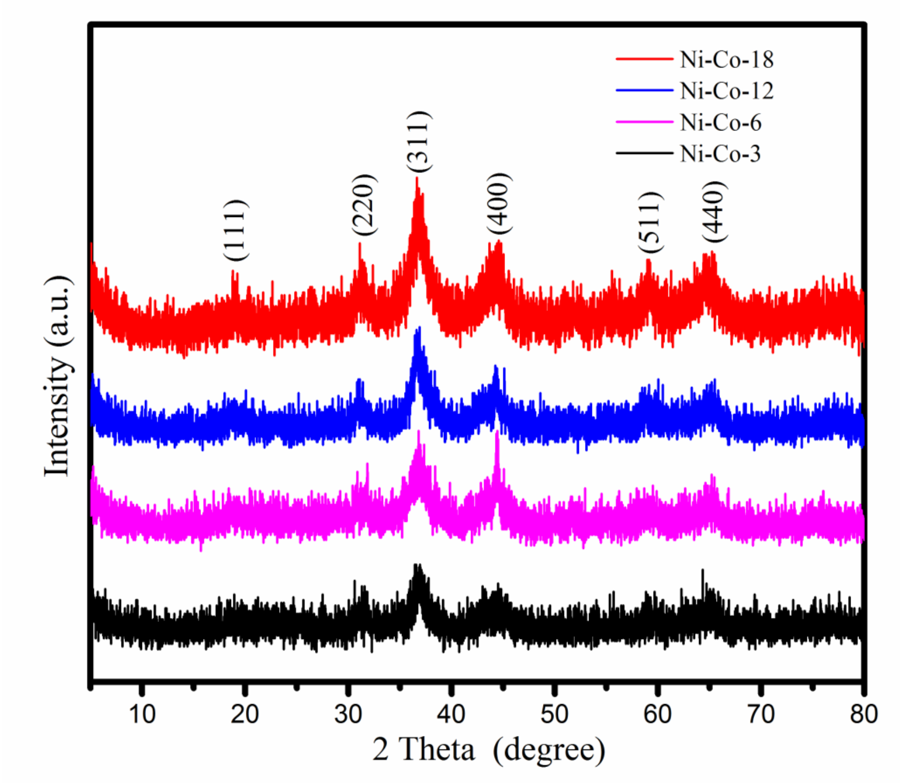

Figure S2. XRD patterns of $NiCo_2O_4$ aerogels after oxidation in air at 300 °C.

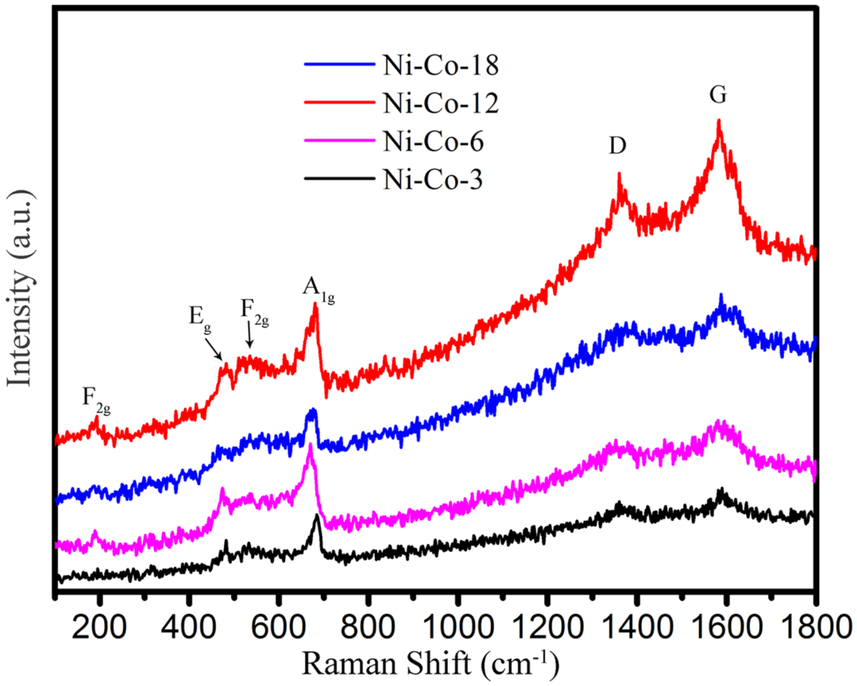

FigureS3. Raman spectra of Ni-Co-3, Ni-Co-6, Ni-Co-12, and Ni-Co-18 aerogels.

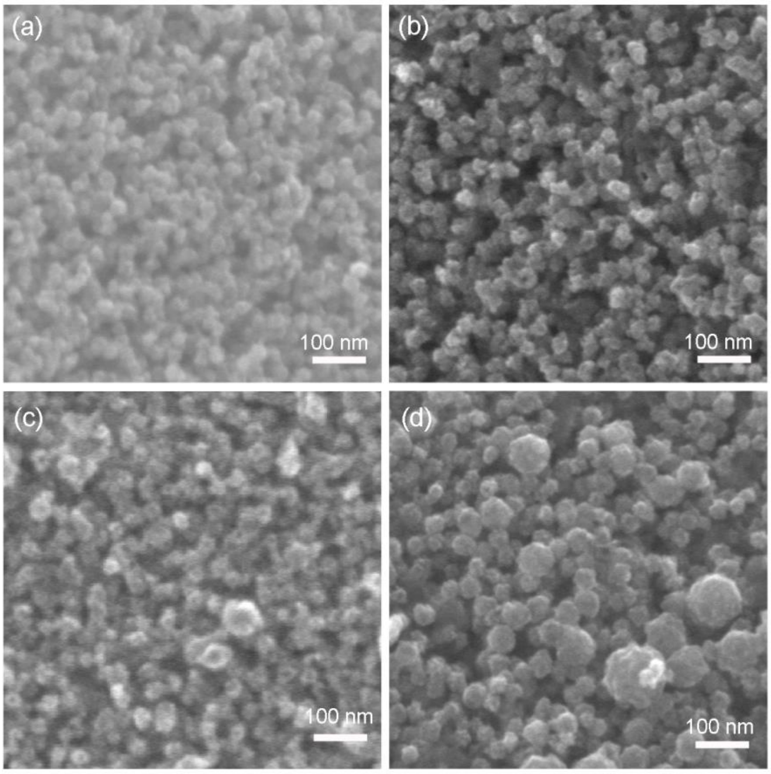

Figure S4. SEM images of Ni-Co-3 (a), Ni-Co-6 (b), Ni-Co-12 (c) and Ni-Co-18 (d) aerogels.

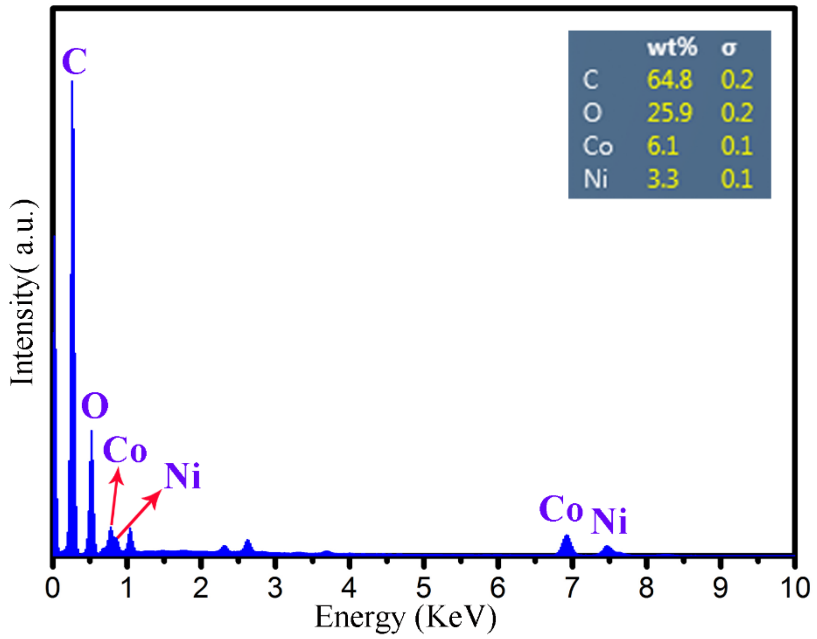

Figure S5. EDS spectrum and the corresponding elemental contents of $NiCo_2O_4$ aerogel.

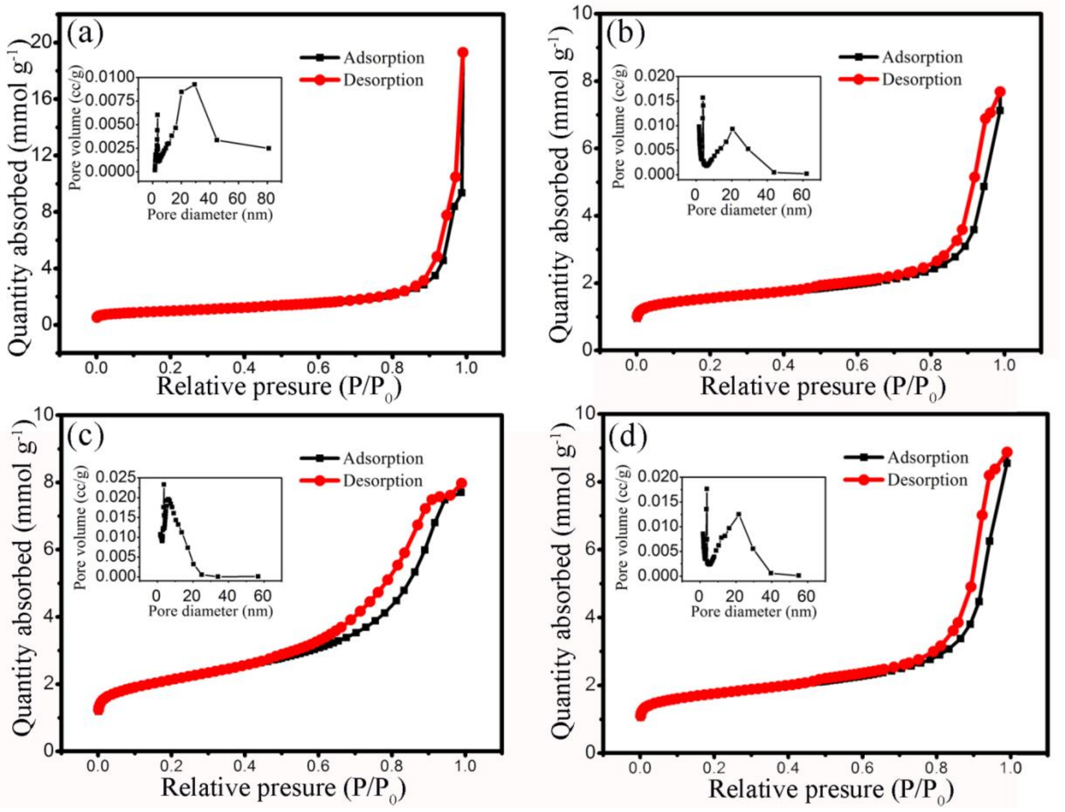

Figure S6. N_2 adsorption/desorption isotherms and pore size distribution curves (insets) of $NiCo_2O_4$ aerogels. (a) Ni-Co-3; (b) Ni-Co-6; (c) Ni-Co-12; (d) Ni-Co-18.

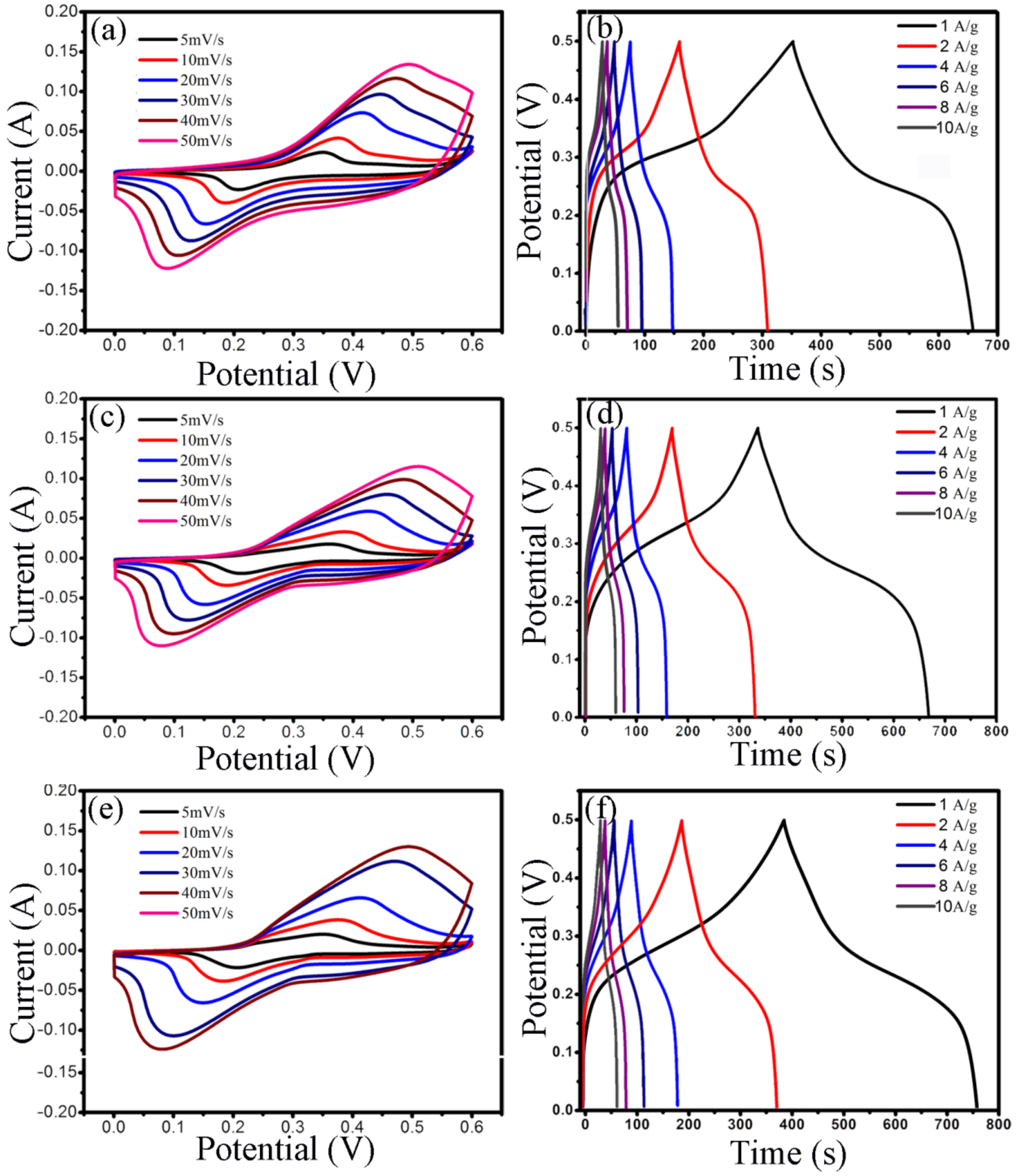

Figure S7. CV and GCD curves of Ni-Co-3 (a, b), Ni-Co-6 (c, d) and Ni-Co-18 (e, f) aerogels.

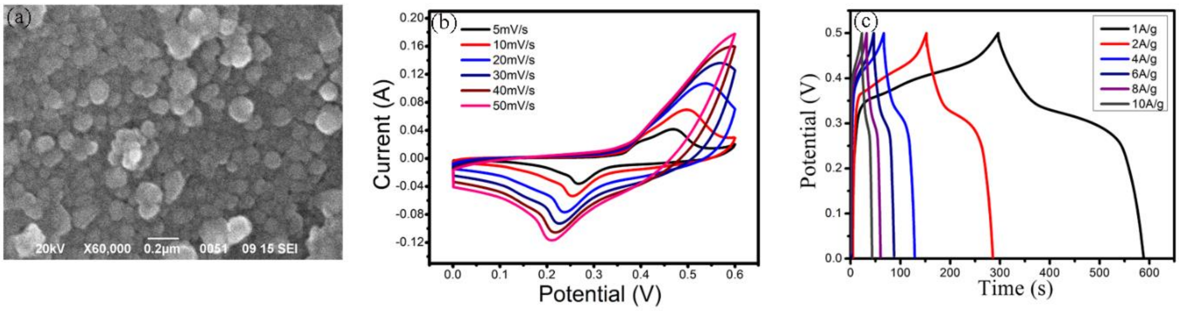

Figure S8. SEM image (a), CV (b) and GCD (c) curves of nonporous $NiCo_2O_4$.

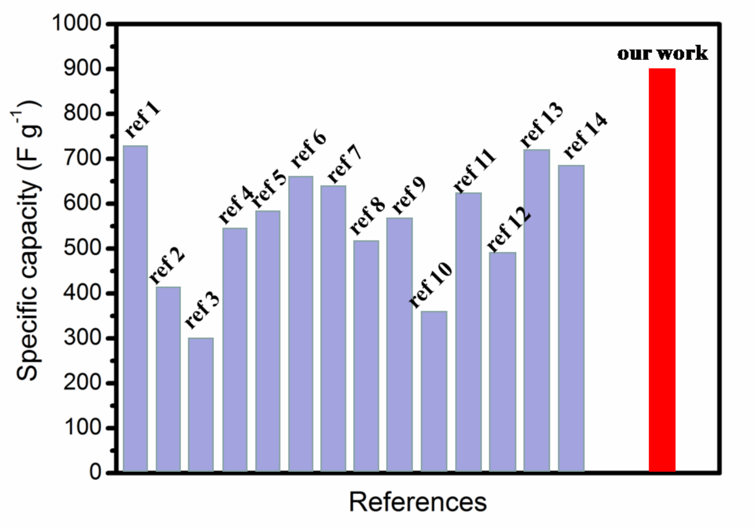

Figure S9. Comparison of the capacitance of the Ni-Co-12 with $NiCo_2O_4$ electrodes recently reported by others at the current density of 2 A g^{-1}.

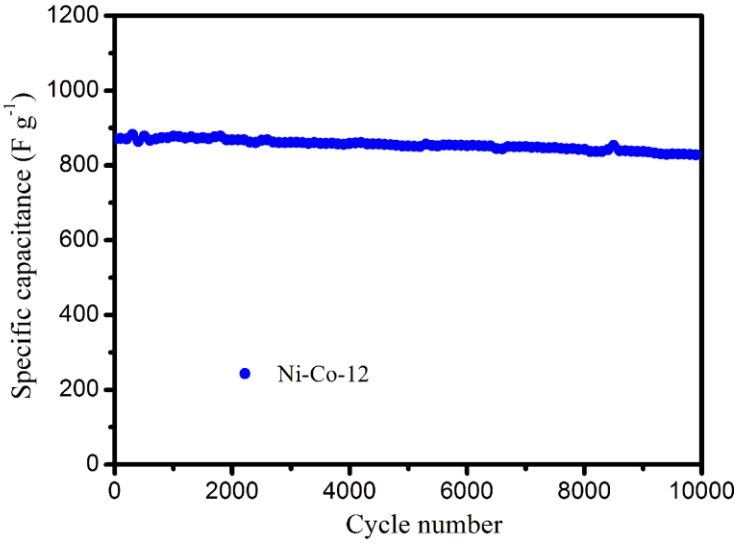

Figure S10. The cycling performance of Ni-Co-12 at a current density of 10 A g^{-1}.

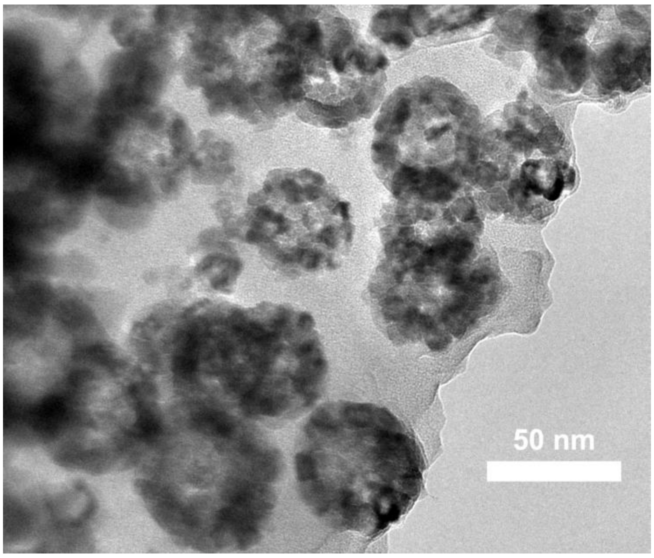

Figure S11. TEM image of Ni-Co-12 after long cycle test.

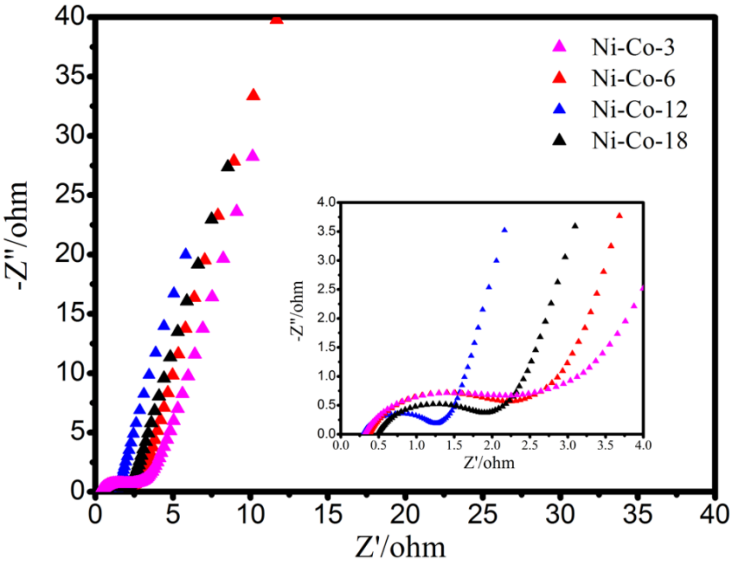

Figure S12. Nyquist plots of Ni-Co-3, Ni-Co-6, Ni-Co-12 and Ni-Co-18.

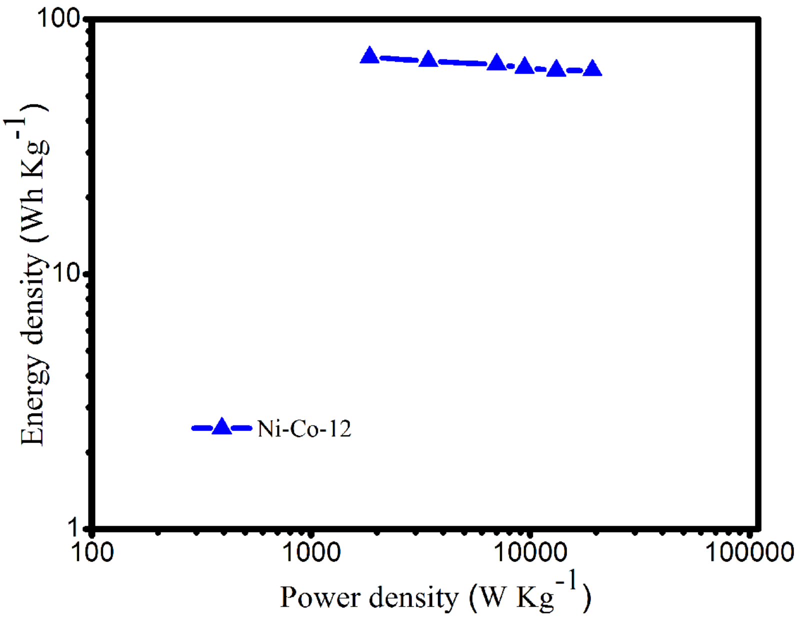

Figure S13. Ragone plot of the HSC.

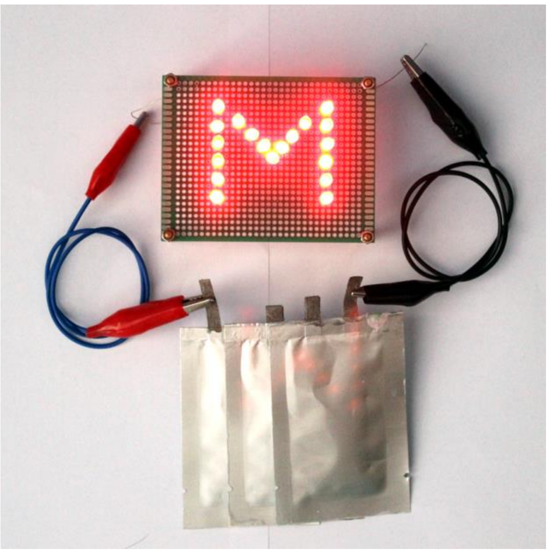

Figure S14. Optical image of the soft package connected in a series to power 19 LED lights.

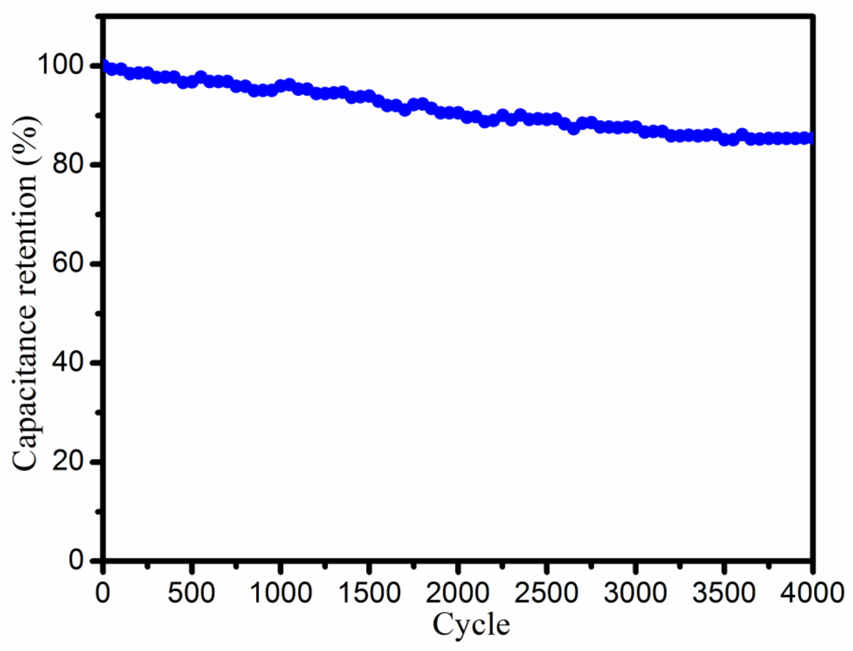

Figure S15. Cycling performance of symmetry supercapacitor device.

Table S1. BET surface areas and pore structures of $NiCo_2O_4$ aerogels.

Samples	BET surface area(m^2 g^{-1})	BJH pore volume (cm^3 g^{-1})	BJH pore size (nm)
Ni-Co-3	77.5	0.007	34.63
Ni-Co-6	121.4	0.027	14.12
Ni-Co-12	167.8	0.022	7.56
Ni-Co-18	137.6	0.028	13.77

Table S2. Specific capacitance values of $NiCo_2O_4$ aerogels at different current densities.

Samples		Current density					
	Units	1A g^{-1}	2A g^{-1}	4A g^{-1}	6A g^{-1}	8A g^{-1}	10A g^{-1}
Ni-Co-3	F/g	617.8	610.4	596.5	587.4	586.9	584.8
Ni-Co-6	F/g	670.4	656.9	644.1	638.8	637.8	635.0
Ni-Co-12	F/g	903.2	898.7	883.0	874.5	870.0	869.0
Ni-Co-18	F/g	763.0	743.2	726.4	706.9	698.3	695.2

References

[1] H. Wang, Q. Gao, L. Jiang, *Small* **2011**, *7*, 2454.

[2] L. Ma, X. Shen, H. Zhou, Z. Ji, K. Chen, G. Zhu, *Chem. Eng. J.* **2015**, *262*, 980.

[3] J. Pu, J. Wang, X. Jin, F. Cui, E. Sheng, Z. Wang, *Electrochim. Acta* **2013**, *106*, 226.

[4] C. Yuan, J. Li, L. Hou, J. Lin, X. Zhang, S. Xiong, *J. Mater. Chem. A* **2013**, *1*, 11145.

[5] C. Yuan, J. Li, L. Hou, J. Lin, G. Pang, L. Zhang, L. Lian, X. Zhang, *Rsc Adv.* **2013**, *3*, 18573.

[6] J. Xiao, S. Yang, *RSC Adv.* **2011**, *1*, 588.

[7] X. Lu, X. Huang, S. Xie, T. Zhai, C. Wang, P. Zhang, M. Yu, W. Li, C. Liang, Y. Tong, *J. Mater. Chem.* **2012**, *22*, 13357.

[8] R. Ding, L. Qi, M. Jia, H. Wang, *J. Appl. Electrochem.* **2013**, *43*, 903.

[9] M. Kuang, W. Zhang, X. L. Guo, L. Yu, Y. X. Zhang, *Ceram. Int.* **2014**, *40*, 10005.

[10] N. Padmanathan, S. Selladurai, *Ionics.* **2013**, *19*, 1535.

[11] Shang. Y. Y, Gai. Y. S, Wang. L. Q, Hao. L, Lv. H. J, Dong. F. Y, Gong. L. Y, *Eur. J. Inorg. Chem.* **2017**, *17*, 2340.

[12] Venkatachalam.V, Alsalme. A, Alghamdi. A, Jayavel. R, *Ionics.* **2017**, *23*, 977.

[13] E. Jokar, A. I. Zad, S. Shahrokhian, *J Solid State Electrochem* **2015**, *19*, 269.

[14]C. R. Zheng, C. B. Cao, R. Chang, J. H. Hou, H. Z. Zhai, *Phys. Chem. Chem. Phys.* ***2016***, *18*, 6268.
